# Single-cell epigenomic variability reveals functional cancer heterogeneity

**DOI:** 10.1186/s13059-016-1133-7

**Published:** 2017-01-24

**Authors:** Ulrike M. Litzenburger, Jason D. Buenrostro, Beijing Wu, Ying Shen, Nathan C. Sheffield, Arwa Kathiria, William J. Greenleaf, Howard Y. Chang

**Affiliations:** 1 Center for Personal Dynamic Regulomes, Stanford University School of Medicine, Stanford, CA 94305 USA; 2 Department of Genetics, Stanford University School of Medicine, Stanford, CA 94305 USA; 3 Department of Applied Physics, Stanford University, Stanford, CA 94035 USA; 4 Broad Institute of MIT and Harvard, Cambridge, MA 02142 USA; 5 Harvard Society of Fellows, Harvard University, Cambridge, MA 02138 USA

**Keywords:** Open chromatin, Gene expression noise, Cancer stem cells

## Abstract

**Background:**

Cell-to-cell heterogeneity is a major driver of cancer evolution, progression, and emergence of drug resistance. Epigenomic variation at the single-cell level can rapidly create cancer heterogeneity but is difficult to detect and assess functionally.

**Results:**

We develop a strategy to bridge the gap between measurement and function in single-cell epigenomics. Using single-cell chromatin accessibility and RNA-seq data in K562 leukemic cells, we identify the cell surface marker CD24 as co-varying with chromatin accessibility changes linked to GATA transcription factors in single cells. Fluorescence-activated cell sorting of CD24 high versus low cells prospectively isolated GATA1 and GATA2 high versus low cells. GATA high versus low cells express differential gene regulatory networks, differential sensitivity to the drug imatinib mesylate, and differential self-renewal capacity. Lineage tracing experiments show that GATA/CD24hi cells have the capability to rapidly reconstitute the heterogeneity within the entire starting population, suggesting that GATA expression levels drive a phenotypically relevant source of epigenomic plasticity.

**Conclusion:**

Single-cell chromatin accessibility can guide prospective characterization of cancer heterogeneity. Epigenomic subpopulations in cancer impact drug sensitivity and the clonal dynamics of cancer evolution.

**Electronic supplementary material:**

The online version of this article (doi:10.1186/s13059-016-1133-7) contains supplementary material, which is available to authorized users.

## Background

Epigenetic aberrations are a key driver of cancer pathogenesis. Altered chromatin states can activate oncogenes and silence tumor suppressor genes, leading to uncontrolled growth and metastasis. In contrast to genetic mutations, epigenetic changes are dynamic and potentially reversible, leading to heterogeneity during development, within tumors, or in response to environmental stimuli, drugs, or diseases [1–4]. Epigenomic variability can arise as cell-to-cell differences in the patterning of DNA methylation, histone modifications, or expression of protein coding genes or noncoding RNAs. This epigenomic variation at the single-cell level can create heterogeneity in cancer. However, the functional relevance of this variation is difficult to assess, often due to a lack of methods capable of quantifying it.

Methods for profiling the epigenomic landscape include bisulfite sequencing for analyzing DNA methylation, DNase-seq and MNase-seq [5–7] for accessibility or nucleosome positioning information, and chromatin immunoprecipitation followed by sequencing (ChIP-seq) for binding sites of individual factors or modified nucleosomes [8, 9]. These methods have proven invaluable for identifying the epigenomic features dictating cell states within large cellular populations but are generally unable to detect single-cell epigenomic cell-to-cell variability. Methods for measuring single-cell gene expression have begun to provide genome-wide measures of cell-to-cell differences; however, these methods provide only an indirect readout of genome-wide epigenomic variance [10, 11]. Recently, single-cell methods for measuring DNA methylation [12, 13], histone modifications [14], and chromatin accessibility have been developed to directly quantify epigenomic variation within cellular populations [15–17]; nevertheless, the functional relevance of this observed epigenomic variability remains to be elucidated.

**Fig. 1:**
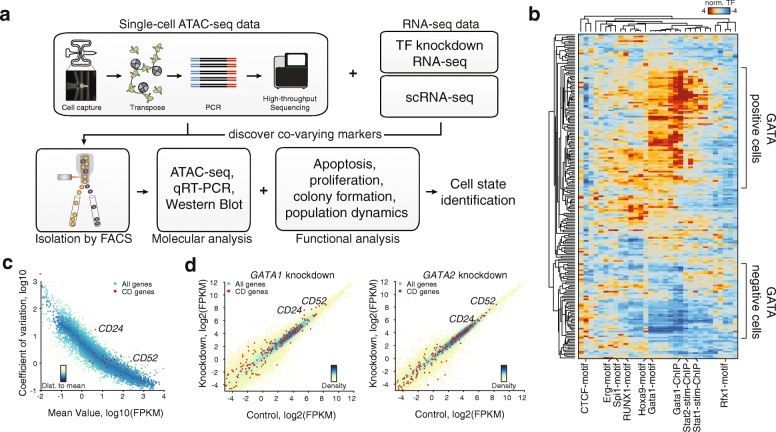
Strategy for identifying a cell surface marker co-varying with identified varying transcription factors. **a** Cartoon illustrating the strategy: single-cell ATAC-seq is followed by sequencing and analysis of cell-to-cell variation, focusing on transcription factor (TF) motifs. RNA-seq and single-cell RNA-seq data are used to correlate cell surface expression with expression of the transcription factor with highest identified variability. The expression of the cell surface protein is subsequently used to isolate subpopulations, which can then be analyzed for molecular and functional characteristics. **b** Hierarchical clustering of cells (*rows*) and high-variance transcription factors (*columns*). Scores represent relative accessibility and are reproduced from Buenrostro et al. [19]. **c** Single-cell RNA-seq data of K562 cells. Coefficient of variation is plotted against the mean FPKM, data points are colored by distance to running mean. *Red dots* indicate *CD* expression markers. **d** Re-analysis of RNA-seq data of *GATA1* and *GATA2* knockdown in K562 cells. Control FPKM is plotted against knockdown FPKM; data points are colored by density. *Red dots* indicate *CD* expression markers. *FACS* fluorescence-activated cell sorting, *qRT-PCR* quantitative reverse transcription PCR

ATAC-seq measures regions of open chromatin using the Tn5-transposase, which preferentially inserts sequencing adapters into accessible chromatin [16]. As applied to single cells [18, 19], this method quantifies cell-to-cell variation in regions of chromatin accessibility. Single cell (sc)ATAC-seq has been used to identify specific transcription factors associated with cell-to-cell regulatory variability, such as GATA1 and GATA2 in K562 cells [19]. While this signal of increased regulatory variation provides a rich platform for hypotheses regarding a potential functional role of GATA factor variation, further experiments are required to identify the phenotypic consequences of this epigenomic variability. Data generated from single-cell techniques like scRNA-seq, scDNA-seq, and scATAC-seq are purely descriptive and require downstream functional validation to link observed heterogeneity to functional subpopulations, such as those with metastatic capabilities or stem cell-like properties that might inform possible treatment strategies. Because most techniques for genomic analysis destroy the cell, it is difficult to combine single-cell approaches with functional cellular assays unless single cells can be identified and sorted using cell surface markers. However, cell surface markers for partitioning cellular populations based on epigenomic state are often unknown. Here we combine scATAC-seq and RNA-seq to identify a potential co-varying surrogate for cell surface markers (Fig. 1a) that enable prospective isolation of relevant subpopulations, allowing downstream functional dissection of the importance of these single-cell observations.

## Results and Discussion

### Selection of cell surface marker co-varying with highly variable motifs identified by scATAC-seq

In previous work, scATAC-seq measurements of K562 chronic myeloid leukemia (CML) cells identified high cell-to-cell variability in the accessibility of the GATA motif (Fig. 1b) [20]. As expected from proliferating cells, we find increased variability within different replication timing domains, representing variable ATAC-seq signal associated with changes in DNA content across the cell cycle. Importantly, the variability in GATA motif accessibility is not influenced by the cell cycle variation [19]. Interestingly, in addition to epigenomic variability associated with GATA binding, we also find high epigenomic variability within transcription factors that are expressed in hematopoietic progenitors, like ERG, HOXA9, SPI1 (PU.1), and RUNX1 [21–24]. We also observe variability associated with STAT1 and STAT2 binding, further reflecting hematopoietic differentiation, as the JAK-STAT pathway is an important regulator enabling cells to respond to interferons and cytokines. In particular, K562 cells contain a BCR-ABL fusion resulting in constitutive STAT activity and ultimately defective erythropoiesis. Furthermore, STAT transcription factors can promote oncogenesis by inducing anti-apoptotic gene expression [25, 26]. These observations suggest that multiple transcription factors involved in regulating the progenitor state significantly vary among K562 cells, pointing to a possible difference in the phenotype of these subpopulations.

Here, we focus on variation in GATA motif accessibility because GATA1 and GATA2 play pivotal roles during erythropoiesis and leukemogenesis [27–30]. Notably, GATA factors have a highly similar binding consensus sequence, WGATAA. Recent genome-wide ChIP-seq analysis using K562 human leukemia cells revealed that 35% of GATA1-binding sites are not occupied by GATA2, while the remaining 65% overlap with GATA2-binding sites [31]. The fact that GATA1 and GATA2 often bind the same subset of genomic locations suggests an underlying mechanism for molecular competition via association and disassociation at the transcription factor binding site. Interestingly, it has also been previously shown that transcription factor crowding on the DNA may increase transcriptional noise through increased variability of the occupancy time of the target sites, leading to cell-to-cell variation [32].

GATA factor interplay is thought to be a common mechanism for controlling developmental processes [33, 34]. During erythropoiesis, GATA2 is expressed prior to GATA1, which suggests that GATA2 binding may promote GATA1 accessibility to GATA motifs. GATA1 occupancy on chromatin has been shown to activate transcription of a differentiation program leading to committed erythroid cells. Here, we test whether the observed variation of DNA accessibility at GATA binding sites resembles functionally distinct developmental cell states. We hypothesize that the accessibility variation results mainly from differential expression levels of GATA in K562 cells (Additional file 1: Figure S1a). To analyze the functional impact of GATA expression and motif accessibility variability, we set out to find a cell surface marker that co-varied with GATA expression levels to allow sorting of live cells from a mixed population for subsequent functional experiments.

Our strategy (Fig. 1a) to identify co-varying transcription factor–cell surface marker pairs starts with analysis of scATAC-seq data, in which we focus on transcription factor motif variability, identifying a transcription factor of interest with variable binding between cells (Fig. 1b). Second, we investigate existing RNA-seq data for cell surface marker expression. scRNA-seq data helps to focus on highly abundant and variably expressed genes. The addition of transcription factor knockdown RNA-seq data allows us to further narrow down candidates. The third phase is the confirmation of co-variation of the transcription factor with the cell surface marker.

Here, K562 scRNA-seq data [35] were analyzed focusing on highly expressed, yet highly variable, cluster of differentiation (“CD”) cell surface genes (red dots in Fig. 1c). In addition, we re-analyze published *GATA1* and *GATA2* knockdown RNA-seq data [36], identifying CD-annotated genes which were both highly expressed and changed expression following *GATA* knockdown in K562 cells (Fig. 1d). Combining both datasets, we identified *CD24*, *CD44*, and *CD52* mRNAs as encoding candidate cell surface genes that were highly variable.

### Validation of a co-varying “surrogate” marker for GATA motif variation

**Fig. 2:**
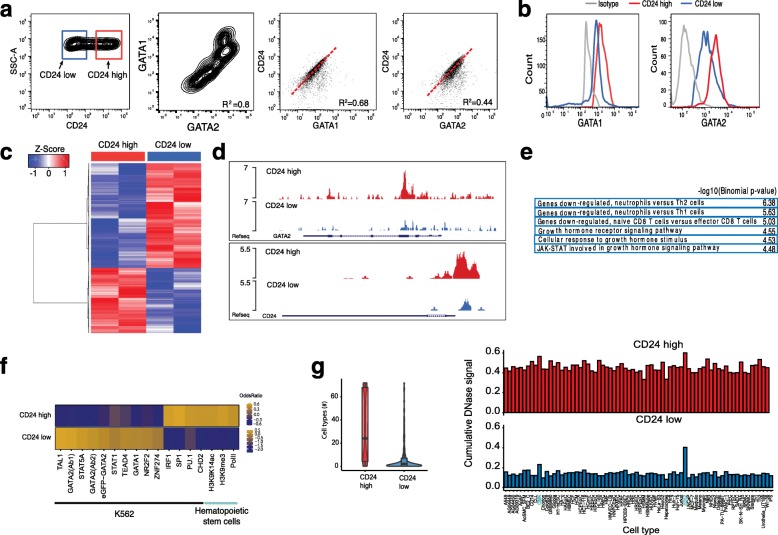
Molecular characteristics of identified subpopulations. **a** Flow cytometric analysis of K562 cells for CD24, GATA1, and GATA2. *Right panels*: CD24 correlates with GATA1 (R^2^ = 0.68) and GATA2 (R^2^ = 0.44). **b** Representative histogram FACS plots of the re-analysis of K562 cells for GATA1 (*left*) and GATA2 (*right*) after sorting for CD24. CD24^hi^ sorted population is labeled *red*, CD24^lo^ sorted population is labeled *blue*, isotype control *gray*. Mean fluorescent intensity (MFI) 2565 for GATA1 high, 2098 for GATA1 low, 2930 for GATA2 high, and 2457 for GATA2 low. **c** ATAC-seq of CD24^hi^ and CD24^lo^ sorted K562 cells (replicates); 2757 peaks are differentially regulated with a fold change of 1.5 and *p* value <0.001. *Blue* represents genomic locations less accessible, *red* locations with higher accessibility compared to the mean of all samples. **d** Representative UCSC genome browser tracks of open chromatin regions in K562 CD24^hi^ sorted cells (*upper track*, *red*) and K562 CD24^lo^ sorted cells (*lower track*, *blue*). Example regions shown are the *GATA2* and *CD24* locus. **e** Gene Ontology term analysis of chromosomal regions, which are more accessible in the CD24^hi^ population. **f** Enrichment of ATAC-seq peaks more open in CD24^hi^ (*top*) or CD24^lo^ (*bottom*) in K562 and hematopoietic stem cell ChIP-seq datasets. Shown are odds ratios calculated using Fisher’s exact test. Values below zero demonstrate de-enrichment (*blue*) and above zero enrichment (*orange*). **g** Overlap of ATAC-seq peaks more accessible in CD24^hi^ (*red*) or CD24^lo^ (*blue*) with DNAse peaks across 72 different cell types. *Left*: Number of cell types with overlap is quantified. *Right*: The different cell types are shown; K562 and CMK leukemia cell lines are highlighted in *green*

To test CD24, CD44, and CD52 as surrogate cell surface markers for GATA variation, we sorted cells with fluorescence-activated cell sorting (FACS). CD44 was only weakly expressed and CD52 did only partially correlate with GATA expression (Additional file 1: Figure S1b). CD24 is expressed and is highly variable in K562 cells (Fig. 2a, left panel); in addition we found two populations, CD24^hi^ (red square) and CD24^lo^ (blue square) (Additional file 1: Figure S1c). GATA1 and GATA2 are also heterogeneously expressed in K562 cells (Fig. 2a, middle panel), with cells expressing low levels of GATA1 also tending to express low levels of GATA2. In a cell with high CD24 expression, GATA1 and GATA2 tend also to be more highly expressed (Fig. 2a, right panels). To further link high expression of CD24 with GATA high cells, cells sorted for CD24 high and low expression were stained and analyzed for GATA. The result shows that in CD24^hi^ cells, protein as well as mRNA levels of GATA1 and GATA2 are higher compared to CD24^lo^ sorted cells (Fig. 2b; Additional file 1: Figure S1d). Notably, expression of phospho-JUN, another transcription factor which displayed high variation in motif accessibility in K562 scATAC-seq experiments [20], does not differ between sorted populations (Additional file 1: Figure S1e). In summary, our data show that CD24 cells are GATA positive and CD24 is thus a surrogate marker for GATA factor expression level in K562 cells.

### Molecular analysis of the identified subpopulations

Focusing on molecular and functional differences of CD24 high versus low K562 subpopulations, we used our CD24 surrogate marker to identify epigenomic differences of the two subpopulations with ATAC-seq. In contrast to other cell lines, the mitochondria are particularly highly represented in K562 cells, resulting in high mitochondrial DNA representation in ATAC-seq libraries. Therefore, we developed an optimized ATAC-seq protocol for K562, which includes an optimized cell lysis and additional nuclei washes prior to transposition, reducing mitochondrial representation from approximately 75 to 35% (see “Methods” for details). Differential peak analysis showed 2757 differentially accessible peaks (fold change (FC) of 1.5, *p* value 0.001; Fig. 2c; Additional file 2: Figure S2a), of which 1698 were more accessible in CD24^lo^ and 1059 more accessible in CD24^hi^ sorted K562 cells. Representative UCSC genome browser tracks of open chromatin regions of CD24^hi^ and CD24^lo^ sorted K562 cells are displayed in Fig. 2d and Additional file 2: Figure S2b. Interestingly, open chromatin regions cluster around transcription start sites in CD24^hi^ (26% in high versus 4% low), whereas in CD24^lo^ K562 cells distal chromatin regions are more accessible (Additional file 2: Figure S2c), suggesting general differential chromatin regulation in these subpopulations. Next we set out to confirm that the differentially accessible sites between CD24^hi^ and CD24^lo^ are functionally relevant. First, we performed Gene Ontology (GO) analysis [37] with all regions more accessible in the CD24^hi^ population, using total accessible locations of K562 cells as background set. These regions are associated with genes involved in neutrophil versus T-cell differentiation, as well as in growth hormone signaling. In particular, STAT signaling is enriched, a signaling pathway involved in CML and BCR-ABL signaling (Fig. 2e) [38, 39]. The resulting gene list was further analyzed with the PANTHER database (http://pantherdb.org), showing the highest biological process GO term enrichment for “regulation of hematopoiesis” (GO:1903706). In contrast, the GO terms resulting from chromatin regions more accessible in CD24^lo^ cells are associated with promoters bound by FOXP3, maturation of monocytes in response to inflammation, MYC overexpression, and genes up-regulated in response to BCR-ABL (Additional file 2: Figure S2d). In addition, we correlated the ATAC-seq peaks more open in CD24^lo^ (1698 genomic regions) as well as those more open in CD24^hi^ (1059 genomic regions) to all available K562 ChIP-seq datasets using LOLA (Locus Overlap Analysis: Enrichment of Genomic Ranges), using total accessible locations of K562 CD24^hi^ and CD24^lo^ cells as background set [40]. Interestingly, ChIP-seq signals of TAL-1, GATA1, and GATA2, factors involved in hematopoietic differentiation [41, 42], are preferentially enriched in accessible locations in CD24^lo^ K562 cells. In CD24^hi^ K562 cells on the other hand, binding sites of the ubiquitous transcription factors SP1, SP2, and CHD2 are enriched, as well as PU.1 sites (Fig. 2f). In addition to the intersection of our ATAC-seq data with ChIP-seq data, we intersected our differential ATAC-seq regions with the regulatory elements database DNAse hypersensitivity data [43]. In line with the previous results, we found high overlap of CD24^lo^ K562 accessible sites with K562 enriched DNAse hypersensitivity clusters, but no enrichment for any specific cell/tissue type for the CD24^hi^ accessible genomic regions (Fig. 2g; Additional file 2: Figure S2e).

These molecular analyses of K562 subpopulations show significantly higher *GATA2* expression in CD24^hi^ cells compared to CD24^lo^ K562 cells (Additional file 1: Figure S1d). However, the CD24^lo^ population exhibits more accessibility at GATA and TAL1 binding sites (Fig. 2f, g; Additional file 2: Figure S2f), transcription factors regulating differentiation into erythrocytes, suggesting that these cells might be more differentiated erythro-leukemic cells. In contrast, the CD24^hi^ K562 population exhibits less erythropoietic-specific transcription factor binding and more accessibility at hematopoietic progenitor maintenance factors, like PU.1 (Fig. 2f, g). PU.1 is a key regulator of hematopoietic differentiation, which is tightly regulated transcriptionally and not expressed in differentiated erythroid or myeloid cells [44] and thus implicates CD24^hi^ as a less differentiated “stem-like” subpopulation. Importantly, *GATA2*, and not *GATA1*, is highly expressed in hematopoietic stem cells, but through erythropoetic differentiation *GATA1* is highly expressed while *GATA2* expression is lost [45]. This “GATA factor switch” is at the center of hematopoietic differentiation and is mediated by GATA factor competition in erythropoetic progenitors, whereby *GATA2* acts as a repressor by inhibiting *GATA1* activation of erythropoetic gene expression [46, 47]. In addition, the over-expression of *GATA2* strongly promotes hematopoietic stem cell self-renewal, altogether implicating GATA2 as a stem-ness factor [48].

We observe on the one hand higher expression of GATA1 and GATA2 in the CD24^hi^ population, an expression signature for more differentiated erythroid cells; on the other hand CD24^hi^ has more accessible binding sites for stem-ness transcription factors. We assume that the high expression of *GATA* in the CD24^hi^ state leads to the overall loss in GATA motif accessibility, whereas GATA motif chromatin accessibility is higher in the more differentiated CD24^lo^ cells, in which *GATA* is also less expressed.

### Functional analysis of the identified subpopulations

**Fig. 3:**
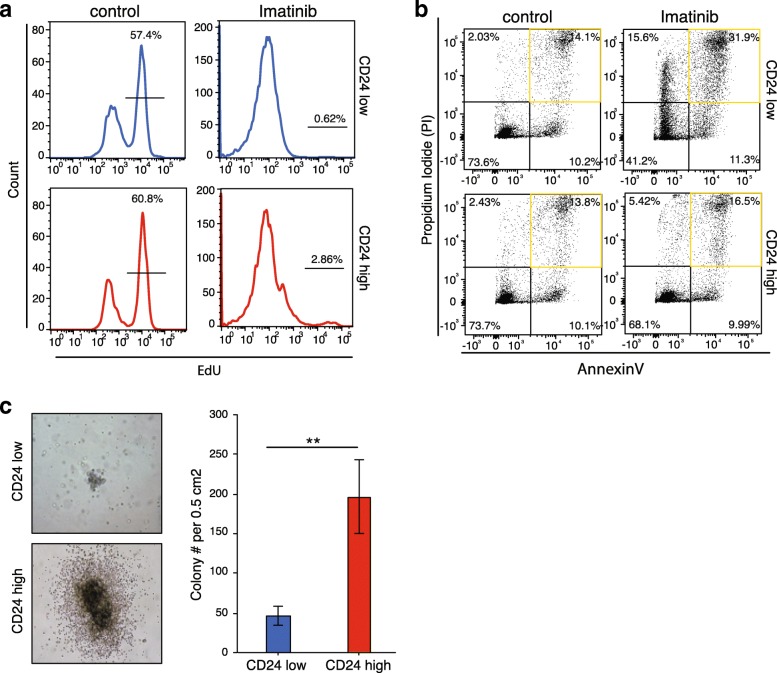
Functional characteristics of identified subpopulations. **a** Proliferation measured by EdU incorporation by K562 cells treated with 1 μM imatinib or DMSO control for 24 h. *Upper panel* (*blue*) shows CD24^lo^ sorted cells, *lower panel* (*red*) shows CD24^hi^ sorted cells. Experiments were performed in triplicate. **b** Annexin–propium iodide FACS of K562 cells treated with 1 μM imatinib or DMSO control for 24 h. *Upper panel* shows CD24^lo^ sorted cells, lower panel shows CD24^hi^ sorted cells. Experiments were performed in triplicate. **c** Colony formation assay of CD24^hi^ and CD24^lo^ K562 cells for 5 days. *Left*: representative microscopy pictures of the colonies formed: CD24^lo^ *upper panel*, CD24^hi^ *lower panel. Right*: Quantification of colonies formed. *Blue* indicates CD24^lo^, *red* CD24^hi^ sorted K562. Experiments were performed in triplicate, *error bars* represent standard error, and asterisks indicate significant difference with *p* value <0.01

Next, we set out to analyze the functional effects of the observed epigenomic variability. The K562 cell line is derived from female human chronic myelogenous leukemia cells, which are positive for the Philadelphia chromosome and bear characteristics of multipotent progenitors [49, 50]. To further elucidate the phenotypic differences of the two subpopulations we treated the CD24^hi^ and CD24^lo^ sorted cells with imatinib mesylate (Gleevec) [51], a BCR-ABL tyrosine kinase inhibitor approved for CML treatment, and observed the effects on proliferation and apoptosis (Fig. 3a, b; Additional file 3: Figure S3a, b). We assayed proliferation by monitoring the incorporation of alkyne-containing thymidine analog EdU (5-ethynyl-2′-deoxyuridine), which is incorporated into DNA during active DNA synthesis [52]. EdU incorporation was significantly inhibited in both subpopulations upon treatment, but 2.9% of CD24^hi^ sorted cells continued proliferating, in contrast to CD24^lo^ sorted cells (Fig. 3a lower right panel; Additional file 3: Figure S3a). To further analyze the differential drug response in more detail, the apoptosis rate of the two cell populations after drug treatment was measured. The percentage of annexin V–propium iodide (PI)-positive cells increased from 14% in control to 32% in the CD24^lo^ population, whereas the number of CD24^hi^ cells undergoing apoptosis was similar (13.8 to 16.5%) (Fig. 3b; Additional file 3: Figure S3b). Therefore, we conclude that CD24^hi^ cells are more resistant to imatinib mesylate treatment than CD24^lo^ cells.

To further support our hypothesis that the CD24^hi^ subpopulation might resemble the more stem cell-like population, whereas the CD24^lo^ subpopulation might be more differentiated, we performed a colony forming cell (CFC) assay, which measures the capacity of single cells to replicate in a semisolid medium, with both sorted subpopulations. The CFC assay allows us to assess the amount of leukemic progenitors within these populations. CD24^hi^ sorted cells formed over fourfold more colonies CD24^lo^ cells (Fig. 3c) and these colonies were generally larger, with a dense core and some outgrowing cells surrounding a ring (Fig. 3c, left panels). These results suggest that the CD24^hi^ population has more progenitor capacity than the CD24^lo^ subpopulation.

We harvested cells from more than four individual colonies or from the whole plate after the CFC assay to further assess their numbers and differentiation states using FACS. We analyzed the CD24 status of the harvested colonies and were surprised to find that the CD24^hi^ subpopulation contained only 30% CD24^hi^ expressing cells; thus, the majority lost their CD24 expression (Additional file 3: Figure S3c). In contrast, the majority of the CD24^lo^ population stayed in the low state, gaining only 6.68% CD24 positive cells. These results suggest that the differentiation state of cancer cells is dynamic, consistent with findings in other cancer stem cell systems [53].

### Epigenomic plasticity of K562 subpopulations

**Fig. 4:**
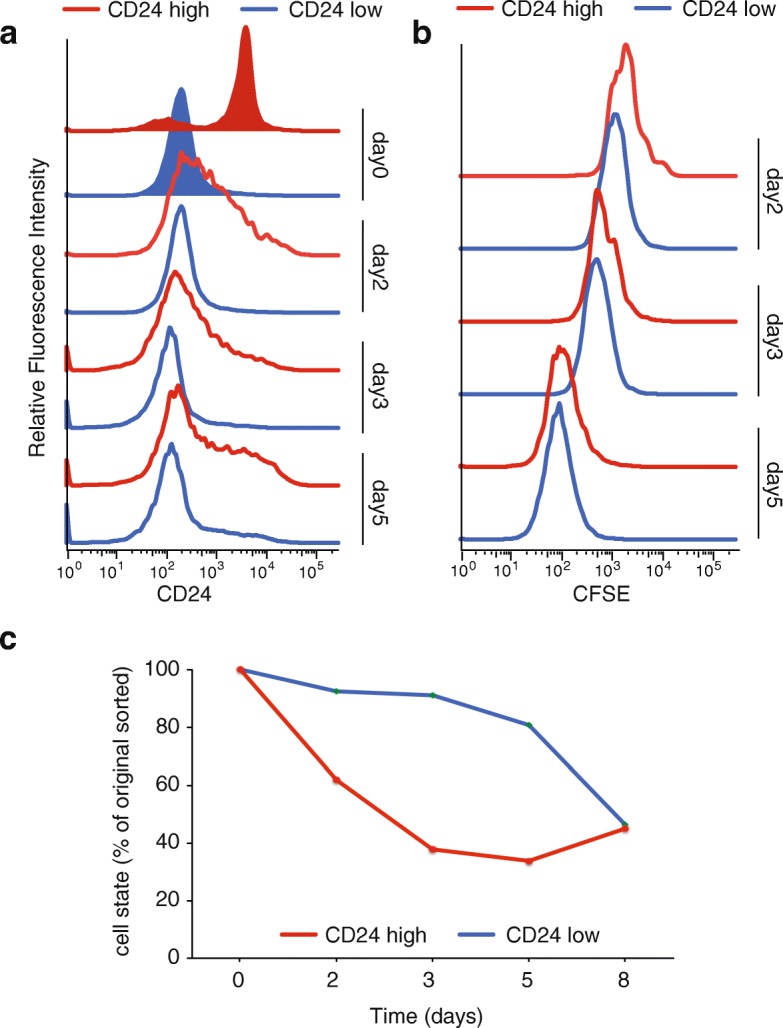
Epigenomic plasticity of K562 subpopulations. **a** FACS analysis of CD24 sorted K562 cells. Shown are the initial sort (*tinted*) and the flow cytometric re-analysis at days 2, 3, and 5. *Blue* indicates CD24^lo^ sorted K562 cells, *red* CD24^hi^ sorted population. **b** Proliferation analysis of K562 sorted subpopulations. After the initial sort CD24^hi^ and CD24^lo^ cells were stained with CFSE and then cultured for 8 days. CFSE fluorescence intensity was measured at days 2, 3, and 5 together with CD24 (**a**). **c** Quantification of the changes in CD24-expressing cells. *Blue*, CD24^lo^; *red*, CD24^hi^

To further investigate these dynamics, K562 cells were sorted for the two subpopulations and immediately stained with the cell tracker 5-(and 6)-carboxyfluorescein diacetate succinimidyl ester (CFSE). CFSE readily crosses intact cell membranes, and after staining cell division can be measured as successive halving of the fluorescence intensity. For five consecutive days CD24 and CFSE signals of the two subpopulations were measured using flow cytometry. Both populations re-established the initial population distribution of CD24^hi^ and CD24^lo^ cells, suggesting that both correspond to metastable, temporally dynamic epigenomic states. We observed a rapid loss of CD24 high expressing cells of the CD24^hi^ sorted subpopulation, whereas the CD24^lo^ subpopulation dynamic changes occurred more slowly (Fig. 4a, c). Both populations proliferated at an equal rate during that time (Fig. 4b). These observations lead to the conclusion that the CD24-GATA-high population is dynamic, and contributes to epigenomic plasticity of K562 cells (Fig. 4c).

To validate the epigenomic plasticity of the identified K562 populations, we cultured the sorted cells (d0) for 5 days (d5) and performed ATAC-seq on CD24 d5 subpopulations. The CD24^hi^ population is able to generate both CD24^hi^ and CD24^lo^ populations within 5 days. We compared the epigenome of the new CD24^hi^-CD24^lo^ populations to each other as well as to the initial sorted (parental) population (Additional file 4: Figure S4a, b): 2884 peaks are differentially accessible in the d5 K562 cells started from the CD24^hi^ population, 1372 more accessible in d5 CD24^hi^, 1512 more accessible in d5 CD24^lo^. The peaks of the parental CD24 sorted K562 cells correlated with the peaks accessible after 5 days with an R of 0.78 and 0.79, respectively (Additional file 4: Figure S4b). Moreover, the new CD24^hi^ and CD24^lo^ populations show the same molecular and phenotypic features as their respective parental line. We analyzed differentially accessible regions between day 5 CD24^lo^ and CD24^hi^ originating from CD24^hi^ using LoLa. The enrichment of accessibility for the respective hematopoietic or more stem factors is in line with what we found with the parental population (Additional file 4: Figure S4c). In addition, we confirmed the functional difference between day 5 CD24^lo^ and CD24^hi^ by apoptosis assay after drug treatment. We sorted day 5 CD24^hi^ and CD24^lo^ K562 cells, treated those with 1 μM imatinib and analyzed them for apoptosis by annexin–PI FACS after 24 h (similar to Fig. 3b). The second-generation CD24^hi^ population cells were less susceptible to the drug (11.1% (standard deviation = 0.84) annexin- and annexin–PI-positive cells compared to 18.5% (standard deviation = 1.56) annexin and annexin–PI positive cells of the second generation CD24^lo^) (Additional file 4: Figure S4d). These results recapitulate the functional heterogeneity found after the first CD24 sort.

## Conclusions

We demonstrate an integrative strategy to prospectively isolate epigenomic subpopulations of cells defined by single-cell chromatin activity. Data mining of available knockdown as well as scRNA-seq data allow correlation of cell surface marker expression with transcription factor variability. scRNA-seq data are generally sparse, making gene–gene correlations, especially of often lowly expressed transcription factors, a particularly difficult task. Our approach, described above, circumvents these issues by looking at functional co-variation using bulk transcription factor knockdowns. This strategy nominates co-varying cell surface markers, which can then be used to identify functional distinct subgroups in cancer cells. A similar approach has been described to resolve heterogeneity within stem cell populations, combining RNA-seq with flow cytometry data [54]. With new genetic perturbation tools like CRISPR [55, 56] and CRISPRi [57], we anticipate this strategy to become more generally applicable and a common tool for single-cell epigenomics. In addition, we anticipate that new high-throughput single-cell genomics methods will be invaluable for efficiently discovering co-varying cell surface markers. Specifically, high-throughput scRNA-seq profiling has been shown to uncover gene-expression networks [58, 59]. Currently, low throughput epigenomics methods preclude identification of the individual regulatory elements within cell populations; however, we anticipate that high-throughput epigenomic methods may enable de novo identification of hidden epigenomic states. This strategy should be broadly applicable to many cancer types and disease states to unravel molecular drivers of epigenomic state and to improve therapeutic targeting.

## Methods

### Cell culture and reagents

K562 (ATCC) chronic myeloid leukemia cells were maintained in Iscove’s modified Dulbecco’s medium (IMDM) containing 10% fetal bovine serum (HyClone, Thermo Scientific) and 1% penicillin streptomycin (Pen/Strep). Cells were maintained at 37 °C and 5% CO_2_ at recommended density and were treated and harvested at mid-log phase for all experiments.

### Drug treatments

K562 cells were treated with 1 μM imatinib mesylate (Gleevec, Cayman Chemicals, Ann Arbor, MI, USA) or DMSO control for 24 h.

### FACS and flow cytometric analysis

In a 1.5 mL tube, cells were washed with ice cold phosphate-buffered saline (PBS). For (CD) cell surface markers, cells were stained with PE-CD24 (#555428, BD Biosciences), or APC-CD44 (#559942, BD Biosciences) or APC-CD52 (Clone HI186, BioLegend) in PBS containing 2 mM EDTA and 0.5% bovine serum albumin (BSA) on ice in the dark for 30 min. For subsequent intracellular staining, cells were fixed in 1% paraformaldehyde (PFA) for 10 min followed by permeabilization using 0.5% TritonX100 in PBS for 10 min at room temperature. Cells were stained with primary antibodies rabbit anti-GATA1 (1:400, Cell Signaling, D52H6), mouse anti-GATA2 (1:100, Abnova, H00002624-M01), rabbit anti phospho c-JUN II (Ser63, Cell Signaling), or mouse or rabbit IgG as isotype control in PBS containing 0.5% TritonX100, 2 mM EDTA and 0.5% BSA (Sigma) for 1 h at room temperature. After washing with staining buffer, cells were labeled with Alexa-conjugated donkey anti-mouse or anti-rabbit Alexa 488 or Alexa 647 antibodies (life technologies) at a dilution of 1:500 for 30 min at room temperature. Finally, cells were washed and sorted for CD24 or analyzed using the BD FACSAriaII.

Flow cytometric analysis and statistics were performed using FlowJo V.10.0.8.

### ATAC-seq

K562 cells were stained and sorted for CD24 as described above. ATAC of 5 × 10^4^ cells was performed as previously described [20], changing the lysis and ATAC conditions slightly. Lysis was performed in 100 μl cold buffer (10 mM Tris-HCl, pH 7.4, 10 mM NaCl, 3 mM MgCl_2_ + 0.1% IGEPAL CA-630 + 0.1% Tween 20), transposition was performed in 50 μl buffer containing 25 μL 2× TD buffer (Illumina #FC-121-1030), 2.5 μL Tn5 transposase (Illumina #FC-121-1030), 22.5 μL nuclease free H_2_O, 0.5 μL Tween-20 (0.1% final), followed by the recommended library preparation protocol. The resulting libraries were quantified and sequencing data were generated on an Illumina HiSeq 4000 that was purchased with funds from NIH under award number S10OD018220.

### Data processing

All ATAC-seq libraries were sequenced using paired-end, dual-index sequencing using 76 × 8 × 8 × 76 cycle reads on a NextSeq. Adapter sequences were trimmed from FASTQs using custom python scripts to enable mapping fragments smaller than 50 bp. Paired-end reads were aligned to hg19 using BOWTIE2 (http://bowtie-bio.sourceforge.net/bowtie2/index.shtml) with the parameter --very-sensitive. Duplicates were removed and library size was estimated using PICARD tools (http://picard.sourceforge.net). Reads were subsequently filtered for alignment quality of > Q30 and were required to be properly paired. Reads mapping to the mitochondria or chromosome Y were removed and not considered. We used MACS2 (http://pypi.python.org/pypi/MACS2) to call all reported ATAC-seq peaks. MACS2 was used with the following parameters (--nomodel --shift 0). Peaks were filtered using the consensus excludable ENCODE blacklist (http://hgdownload.cse.ucsc.edu/goldenPath/hg19/encodeDCC/wgEncodeMapability/) and a custom blacklist designed to remove high-signal-causing repeats and mitochondrial homologues. Using the filtered peak set, peak summits were extended ±250 bps. The top 50,000 non-overlapping 500-bp summits, which we refer to as accessibility peaks, were used for all downstream analysis.

Peaks from all samples were merged and normalized. For differentially accessible peaks a cutoff of 1.5-fold change and *p* value <0.01 between CD24^hi^ and CD24^lo^ were used. For ATAC-seq peak–ChIPseq and DNAse-seq correlation analysis we used the LOLA bioconductor package with all K562 peaks from these ATAC-seq experiments as background set. For enrichment of GATA2-bound motifs in ATAC-seq peaks, ChIP-seq dataset GSM935373 was intersected with ATAC-seq peaks.

GO term analysis was performed using GREAT (http://great.stanford.edu) [37].

K562 CD24 sorted ATAC-seq data from day 0 and day 5 have been deposited in the Gene Expression Omnibus (GEO) with accession GSE76224.

### Quantitative RT-PCR

Total RNA was isolated with an RNeasy isolation kit (Qiagen) and cDNA was synthesized using the Superscript III First Strand synthesis kit according to the manufacturer’s instructions (Invitrogen). qRT-PCR reactions were performed in a Roche Lightcycler 480 using 2× Brilliant II SYBR QRT-PCR Master Mix from Agilent according to standard protocols. All primers were separated by at least one intron on the genomic DNA to exclude amplification of genomic DNA. PCR reactions were checked by including no-RT controls, by omission of templates, and by examining melting curves. Standard curves were generated for each gene. Relative quantification of gene expression was determined by comparison of threshold values. All samples were analyzed in duplicate in two different dilutions. All results were normalized to actin. All experiments were performed in biological triplicates.

Primer sequences were (5′–3′ forward, reverse): actin, CCGGCTTCGCGGGCGACG, TCCCGGCCAGCCAGGTCC; *GATA1*, TGCTCTGGTGTCCTCCACAC, TGGGAGAGGAATAGGCTGCT; *GATA2*, AGCGTCTCCAGCCTCATCTTCCGCG, CGAGTCTTGCTGCGCCTGCTT.

### Proliferation measurements

K562 cells were sorted for CD24 and cultured in the presence of 1 μM imatinib mesylate or DMSO for 24 h before proliferation analysis. EdU (10 μM) was added directly to the media for 4 h before cells were harvested. After that, cells were fixed and stained according to the manufacturer’s protocol (Click-iT EdU kit #C10340, Invitrogen). Briefly, cells were fixed with 3.7% formaldehyde for 15 min and permeabilized using 0.5% Triton X-100 in PBS for 20 min at room temperature. Incorporation of EdU was observed by incubating fixed cells with 2% BSA in PBS for 30 min and Alexa fluor 647 for a further 30 min under Cu(I)-catalyzed click reaction conditions, as described by the manufacturer. Cells were washed with PBS and counterstained with DAPI in PBS right before flow cytometric analysis using the BD FACSAriaII.

Experiments were performed in triplicate; the standard 10,000 cells per gate were recorded and analyzed.

### Apoptosis measurements

K562 cells were sorted for CD24 and cultured in the presence of 1 μM imatinib mesylate or DMSO for 24 h before proliferation analysis. Cells were washed with cold PBS containing 0.5% BSA and then resuspended in Annexin V Binding Buffer (BioLegend, #422201). Cells were then incubated for 15 min with 5 μl of FITC annexin V (BioLegend, #640906) and 10 μl of 1 mg/ml PI solution (BioLegend, #421301) at room temperature in the dark. Apoptosis was measured by flow cytometry using the BD FACSAriaII.

Experiments were performed in triplicate; the standard 10,000 cells per gate were recorded and analyzed.

### Colony formation assay

K562 cells were sorted for CD24. Immediately after sorting, 500 cells in 0.5 ml medium were added to 3 ml methylcellulose-based media (HSC002, R&D Systems). Using a 10 ml syringe and a 16 gauge needle, 1 ml of this mixture was added to a 35-mm dish, which was then placed in a 15-cm dish filled with water to maintain the humidity necessary for colony formation. After 10 days, colonies were counted on a grid using a light microscope. After that, methylcellulose was dissolved in media to make a single-cell suspension. Cells were washed and stained as described above for flow cytometric analysis of CD24 expression using the BD FACSAriaII. Experiments were performed in triplicate.

### Cell tracing experiments (CFSE staining)

K562 cells were sorted for CD24. Immediately after sorting, 200,000 cells of the high- and low-sorted population were stained with 5 μM CFSE (Cell Trace Proliferation Kit, Life Technologies) according to the manufacturer’s protocol. Cell proliferation (CFSE dilution) and CD24 surface expression were analyzed every 24 h for 8 days using the BD FACSAriaII.

Experiments were performed in triplicate; the standard 10,000 cells per gate were recorded and analyzed.

## Additional files

Additional file 1: Figure S1. Molecular characteristics of identified subpopulations. a *Left*: QRT-PCR of *GATA1* and *GATA2* in K562 cells measured relative to *ACTIN. Error bars* represent standard error. *Right*: Representative FACS analysis of K562 cells stained for GATA1 and GATA2. b Histograms showing expression (fluorescent intensity) of CD44 (*light blue*) and CD52 (*dark blue*) in K562 cells. c Dot plots displaying the gating strategy for sorting CD24^hi^ and CD24^lo^ expressing K562 cells. d Expression analysis of *GATA1* and *GATA2* in CD24 sorted K562 cells measured by qRT-PCR relative to *ACTIN*. **P* value <0.05 (t-test), *error bars* represent standard error. e Representative FACS analysis of CD24^hi^ and CD24^lo^ sorted K562 cells, stained after sort for pJUN. Mean fluorescent intensity (MFI) was 152 (high), and 137 (low). (PDF 616 kb)
Additional file 2: Figure S2. Molecular characteristics of identified subpopulations. a Heatmap of the correlation coefficient of K562 CD24 sorted ATAC-seq samples using Spearman correlation. b UCSC tracks showing examples of open chromatin in CD24^lo^ (*blue*) and CD24^hi^ (*red*) K562 cells. *Top*: *JUN* locus, which is more accessible in CD24^lo^. *Bottom*: *SOD1* locus, which is equally accessible in both subpopulations. c Bar plot illustrating the distribution of ATAC-seq peaks across genomic locations; promoter proximal (*light blue*) and distal (*dark blue*). The difference in promoter accessibility between CD24^hi^ and CD24^lo^ K562 cells is significant (using Chi-squared), *p* < 0.001. d Gene Ontology terms for accessible chromatin locations in CD24^lo^ cells. e Q-Q plot illustrating the differences in the overlap of ENCODE DNAse-seq peaks and ATAC-seq peaks of CD24^lo^ and CD24^hi^ populations (shown in Fig. 2g). *P* values for Wilcox (*WX*), t-test (*TT*), and Komorov–Smirnov (*KS*). f Enrichment of GATA2 ChIP-seq binding sites in CD24^hi^ and CD24^lo^ K562 ATAC-seq peaks. (PDF 507 kb)
Additional file 3: Figure S3. Functional characteristics of identified subpopulations. a Quantification of EdU incorporation as a measurement of proliferation. Experiments were done in triplicate; *asterisk* indicates significance, calculated using t-test, *p* value <0.05. b Quantification of apoptosis of K562 cells treated with 1 μM imatinib or DMSO control for 24 h. AnnexinV–PI negative cells are counted as live, annexin V-positive–PI-negative cells as early apoptotic and annexin V–PI double positive as late apoptotic. Experiments were done in triplicate; *asterisk* indicates significance, calculated using t-test, *p* value <0.01. *Error bars* represent standard error. c Representative FACS analysis of CD24^hi^ and CD24^lo^ sorted K562 cells for CD24 expression after 5-day colony formation assay. (PDF 138 kb)
Additional file 4: Figure S4. Molecular and functional analysis of epigenetic dynamics. a Heat map of differentially accessible ATAC-seq peaks of day 5 CD24^hi^ and CD24^lo^ K562 cells (replicates). CD24^hi^ as parental line: 2884 peaks are differentially accessible, 1372 more accessible in day 5 CD24^hi^, 1512 more accessible in day 5 CD24^lo^. Fold change of 1.5 and *p* value <0.001. *Blue* represents genomic locations less accessible, *red* locations with higher accessibility compared to the mean of all samples. b Spearman correlation of day 5 K562 CD24^hi^ (*top*) and CD24^lo^ (*bottom*) ATAC-seq peaks (log counts) and day 0 (parental) ATAC-seq peaks. R = 0.78 and 0.79, respectively, *p* value of the correlation <2.2e-16 and 0.0012, respectively. c LoLa analysis of differentially accessible peaks of new (day 5) CD24^hi^ and CD24^lo^. List of the highest odds ratios in untreated K562 datasets. d AnnexinV–PI apoptosis FACS of day 5 CD24^hi^ and CD24^lo^ 24 h after imatinib treatment. Bar plots demonstrate percentage of total cells in different phases of apoptosis. *Red* bars represent cells originating from CD24^hi^, *blue* bars demonstrate CD24^lo^ descendants. N = 3. (PDF 7944 kb)

## Abbreviations

ATAC-seq: Assay for transposase-accessible chromatin with high-throughput sequencing
BSA: Bovine serum albumin
CFC: Colony formation assay
CFSE: Carboxyfluorescein succinimidyl ester
ChIP: Chromatin immunoprecipitation
CML: Chronic myeloid leukemia
EdU: 5-ethynyl-2′-deoxyuridine
FACS: Fluorescence-activated cell sorting
GO: Gene Ontology
PBS: Phosphate-buffered saline
PI: Propidium iodide
qRT-PCR: Quantitative reverse transcription polymerase chain reaction
sc: Single-cell
scATAC-seq: Single-cell assay for transposase-accessible chromatin with high-throughput sequencing
scRNA-seq: Single-cell RNA sequencing
