# Supplementary figures and images for: Single-cell epigenomic variability reveals functional cancer heterogeneity
Source: Genome Biol. 2017 Jan 24;18:15. doi: 10.1186/s13059-016-1133-7 (PMC5259890; doi:10.1186/s13059-016-1133-7)

A

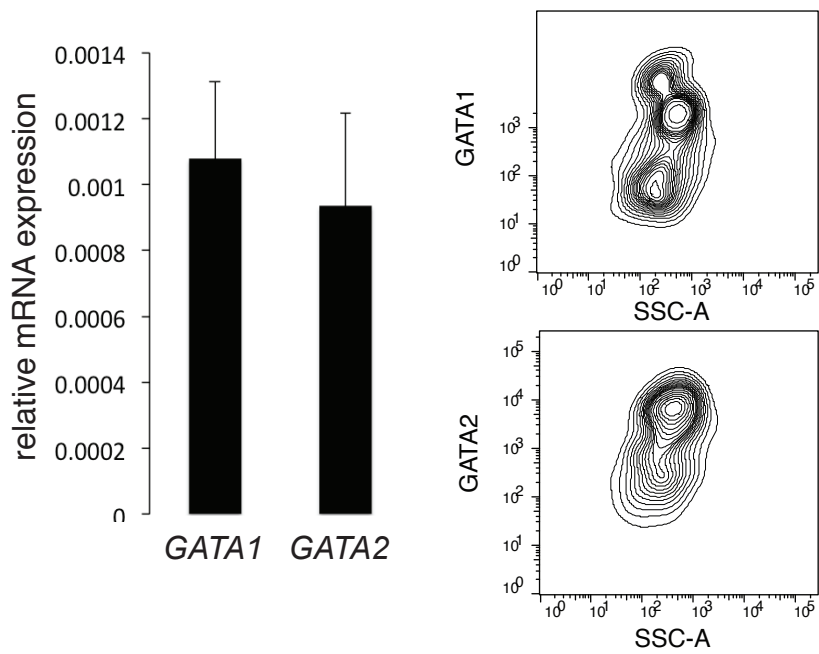

B

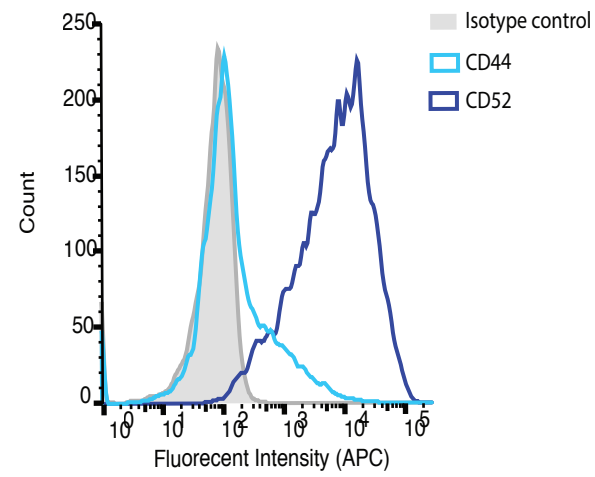

C

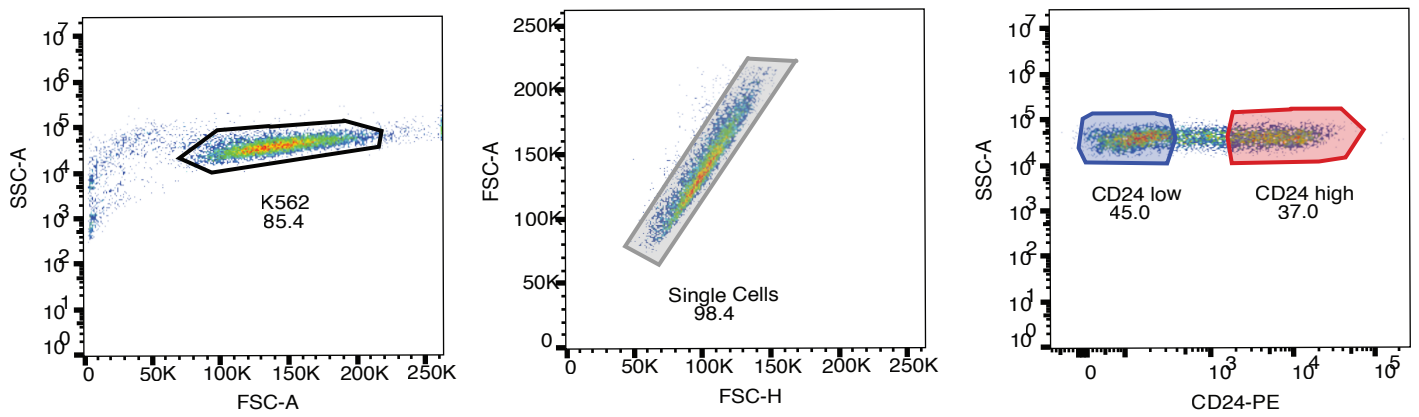

D

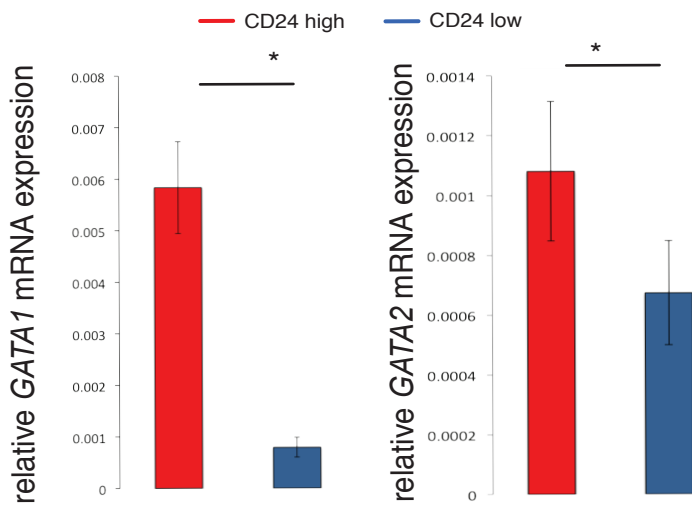

E

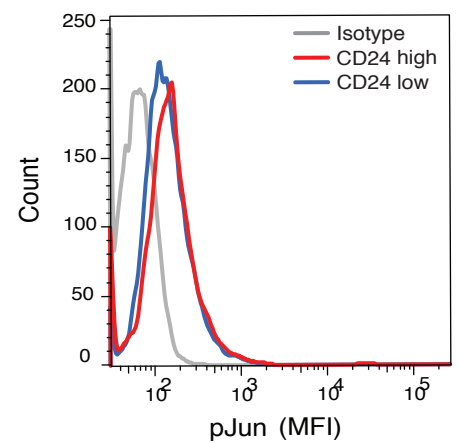

Supplement: Additional file 1: Figure S1. — Molecular characteristics of identified subpopulations. a Left: QRT-PCR of GATA1 and GATA2 in K562 cells measured relative to ACTIN. Error bars represent standard error. Right: Representative FACS analysis of K562 cells stained for GATA1 and GATA2. b Histograms showing expression (fluorescent intensity) of CD44 (light blue) and CD52 (dark blue) in K562 cells. c Dot plots displaying the gating strategy for sorting CD24hi and CD24lo expressing K562 cells. d Expression analysis of GATA1 and GATA2 in CD24 sorted K562 cells measured by qRT-PCR relative to ACTIN. *P value <0.05 (t-test), error bars represent standard error. e Representative FACS analysis of CD24hi and CD24lo sorted K562 cells, stained after sort for pJUN. Mean fluorescent intensity (MFI) was 152 (high), and 137 (low). (PDF 616 kb) [file 13059_2016_1133_MOESM1_ESM.pdf]

**A**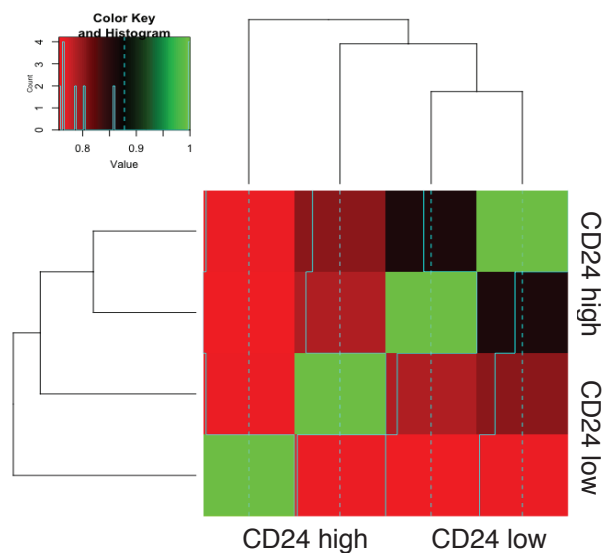**B**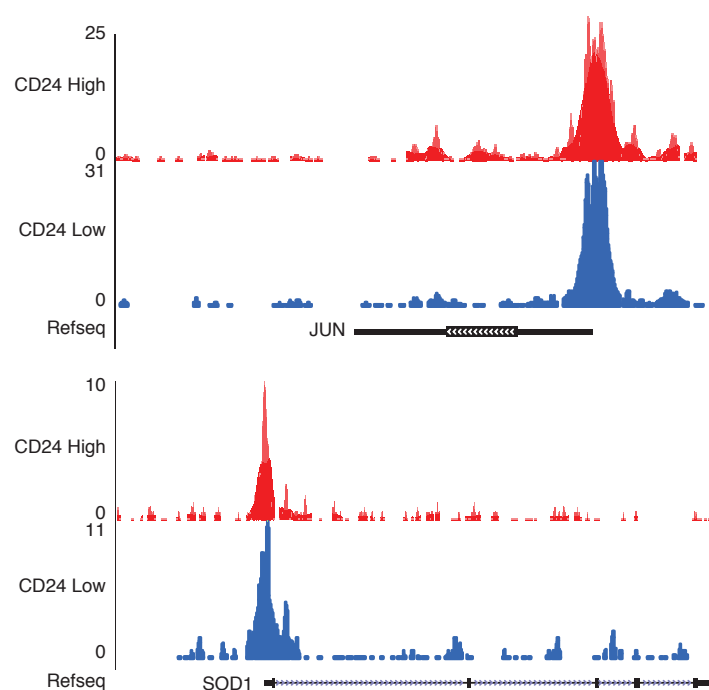**C**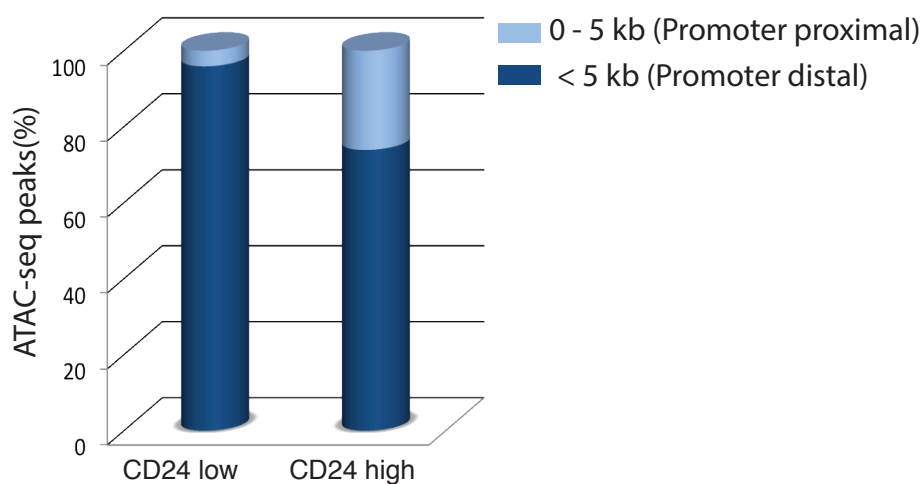**D**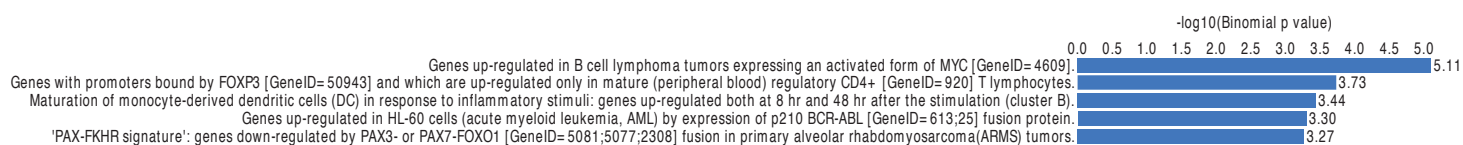**E**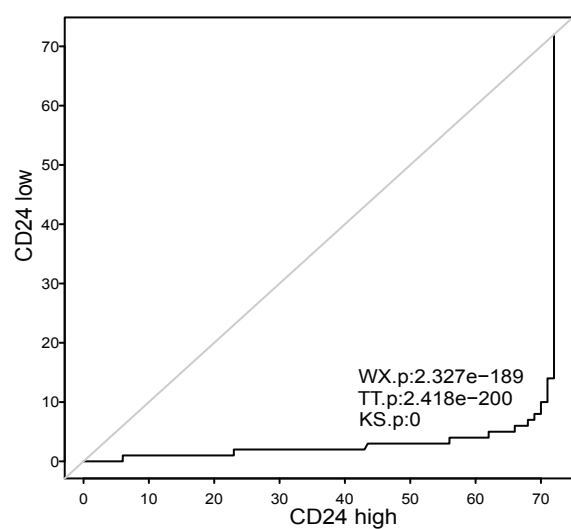**F**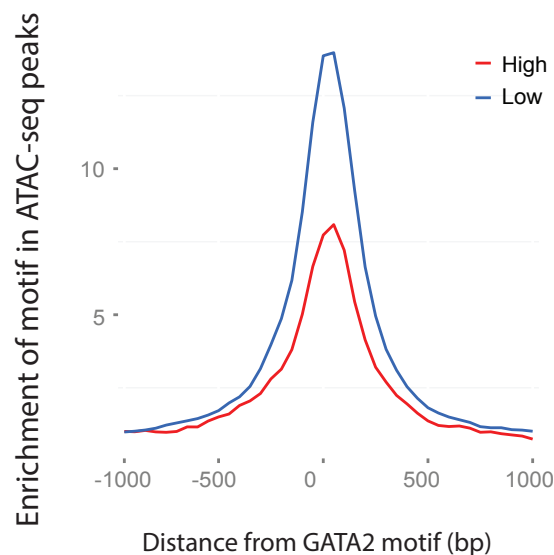

Supplement: Additional file 2: Figure S2. — Molecular characteristics of identified subpopulations. a Heatmap of the correlation coefficient of K562 CD24 sorted ATAC-seq samples using Spearman correlation. b UCSC tracks showing examples of open chromatin in CD24lo (blue) and CD24hi (red) K562 cells. Top: JUN locus, which is more accessible in CD24lo. Bottom: SOD1 locus, which is equally accessible in both subpopulations. c Bar plot illustrating the distribution of ATAC-seq peaks across genomic locations; promoter proximal (light blue) and distal (dark blue). The difference in promoter accessibility between CD24hi and CD24lo K562 cells is significant (using Chi-squared), p < 0.001. d Gene Ontology terms for accessible chromatin locations in CD24lo cells. e Q-Q plot illustrating the differences in the overlap of ENCODE DNAse-seq peaks and ATAC-seq peaks of CD24lo and CD24hi populations (shown in Fig. 2g). P values for Wilcox (WX), t-test (TT), and Komorov–Smirnov (KS). f Enrichment of GATA2 ChIP-seq binding sites in CD24hi and CD24lo K562 ATAC-seq peaks. (PDF 507 kb) [file 13059_2016_1133_MOESM2_ESM.pdf]

**A**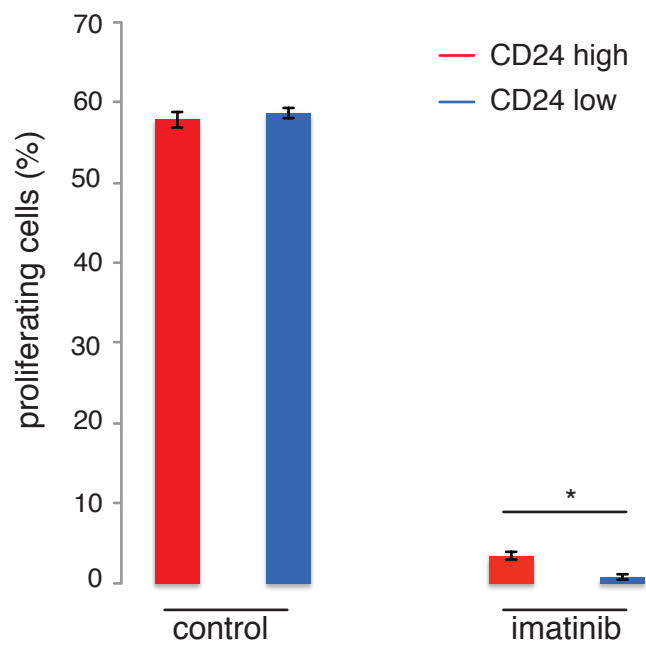**B**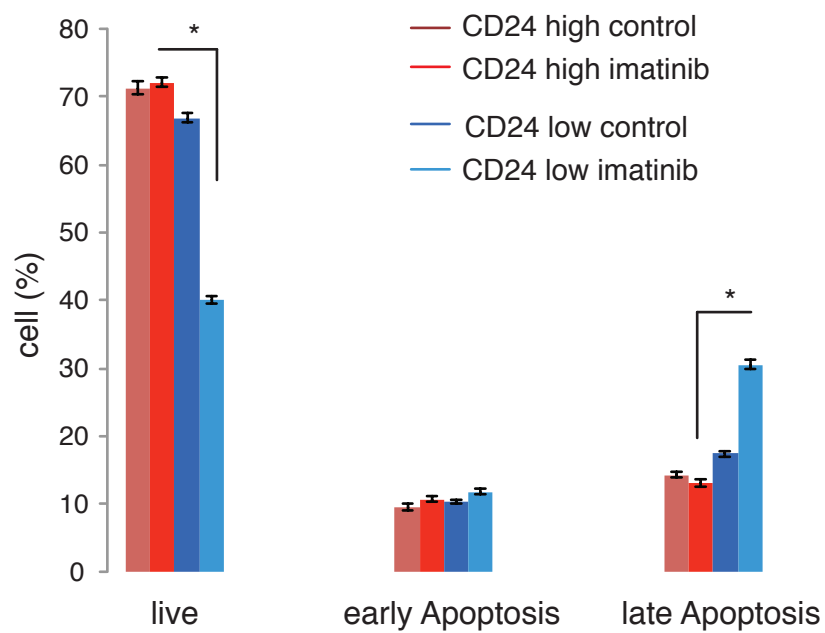**C**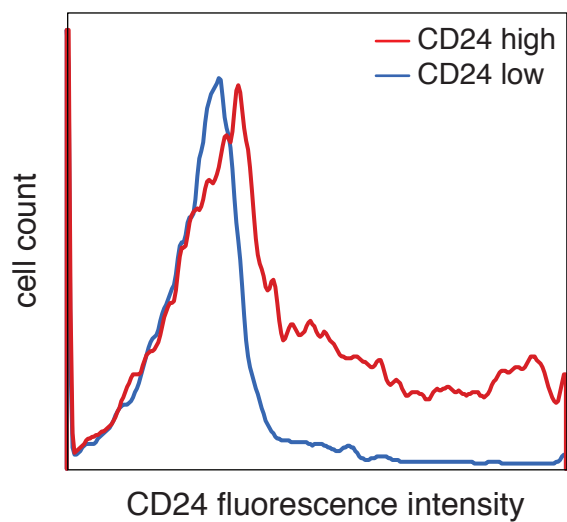

Supplement: Additional file 3: Figure S3. — Functional characteristics of identified subpopulations. a Quantification of EdU incorporation as a measurement of proliferation. Experiments were done in triplicate; asterisk indicates significance, calculated using t-test, p value <0.05. b Quantification of apoptosis of K562 cells treated with 1 μM imatinib or DMSO control for 24 h. AnnexinV–PI negative cells are counted as live, annexin V-positive–PI-negative cells as early apoptotic and annexin V–PI double positive as late apoptotic. Experiments were done in triplicate; asterisk indicates significance, calculated using t-test, p value <0.01. Error bars represent standard error. c Representative FACS analysis of CD24hi and CD24lo sorted K562 cells for CD24 expression after 5-day colony formation assay. (PDF 138 kb) [file 13059_2016_1133_MOESM3_ESM.pdf]
