# Supplementary material for: Single-cell epigenomic variability reveals functional cancer heterogeneity
Source: Genome Biol. 2017 Jan 24;18:15. doi: 10.1186/s13059-016-1133-7 (PMC5259890; doi:10.1186/s13059-016-1133-7)
Supplement: Additional file 4: Figure S4. — Molecular and functional analysis of epigenetic dynamics. a Heat map of differentially accessible ATAC-seq peaks of day 5 CD24hi and CD24lo K562 cells (replicates). CD24hi as parental line: 2884 peaks are differentially accessible, 1372 more accessible in day 5 CD24hi, 1512 more accessible in day 5 CD24lo. Fold change of 1.5 and p value <0.001. Blue represents genomic locations less accessible, red locations with higher accessibility compared to the mean of all samples. b Spearman correlation of day 5 K562 CD24hi (top) and CD24lo (bottom) ATAC-seq peaks (log counts) and day 0 (parental) ATAC-seq peaks. R = 0.78 and 0.79, respectively, p value of the correlation <2.2e-16 and 0.0012, respectively. c LoLa analysis of differentially accessible peaks of new (day 5) CD24hi and CD24lo. List of the highest odds ratios in untreated K562 datasets. d AnnexinV–PI apoptosis FACS of day 5 CD24hi and CD24lo 24 h after imatinib treatment. Bar plots demonstrate percentage of total cells in different phases of apoptosis. Red bars represent cells originating from CD24hi, blue bars demonstrate CD24lo descendants. N = 3. (PDF 7944 kb) [file 13059_2016_1133_MOESM4_ESM.pdf]

A

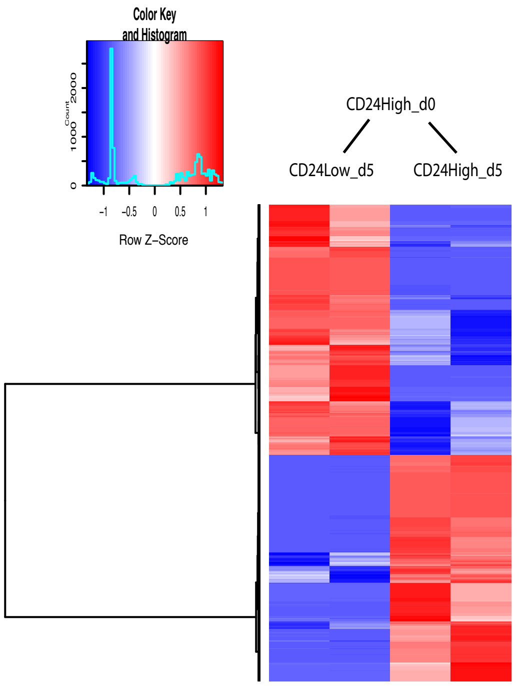

B

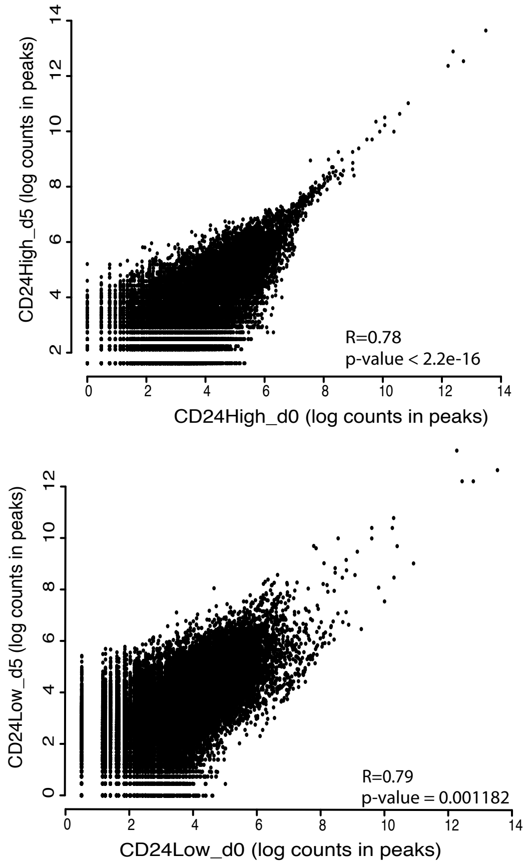

C

cell type	factor	odds ratio
K562	GATA1	3.178284318
K562	GATA-2	1.69448295
K562	c-Jun	1.884249584
K562	eGFP-FOS	1.59137467
Proerythroblast	TAL1	2.770485032
Erythroblast	SMAD1	6.298336296

cell type	factor	odds ratio
K562	TBP	2.945140764
K562	PHF8	2.717283073
K562	ELF1	2.846414281
K562	Pol2	2.676088652
K562	Egr-1	2.545006798
K562	SIRT6	3.260518086

D

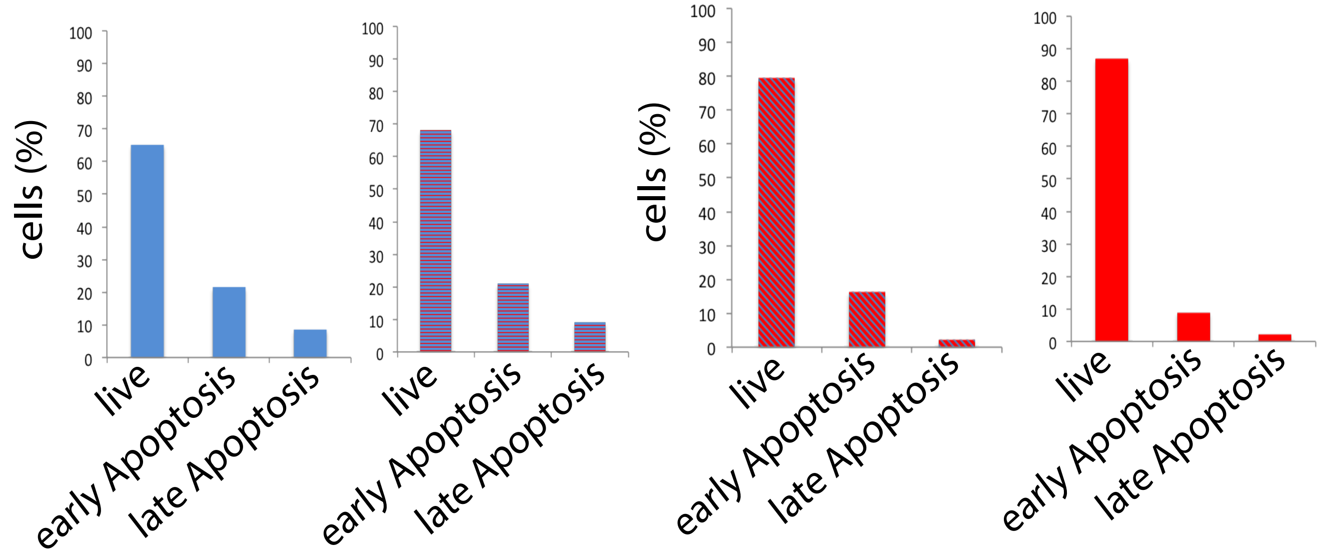

Supplementary Figure4
